# Markers of chronic disease risk in term low birthweight Indian children aged 8–14 years

**DOI:** 10.3389/fped.2024.1339808

**Published:** 2024-08-29

**Authors:** Yamini Gusain, Anku Malik, Suzanne Filteau, Renuka Pathak, Harshpal Singh Sachdev, Geeta Trilok-Kumar

**Affiliations:** ^1^ Department of Food and Nutrition, Institute of Home Economics, University of Delhi, New Delhi, India; ^2^ Department of Population Health, Faculty of Epidemiology and Population Health, London School of Hygiene and Tropical Medicine, London, United Kingdom; ^3^ Department of Pediatrics and Clinical Epidemiology, Sitaram Bhartia Institute of Science and Research, New Delhi, India; ^4^ Koita Centre for Digital Health, Ashoka University, Panipat, India

**Keywords:** chronic disease, biomarkers, low birth weight, children, India

## Abstract

**Background:**

Low birth weight (LBW) is a public health problem in India with consequences in the short and long term. It increases the risk of obesity and its related comorbidities including type 2 diabetes and cardiovascular disease (CVD) in later life. This study aimed to assess the risk markers of chronic disease in term born low birthweight Indian children aged 8–14 years.

**Methods:**

This was a cross-sectional follow-up of LBW children from DIViDS (Delhi Infant Vitamin D Supplementation) cohort and involved assessment of their anthropometric measurements, body composition, levels of adipokines and biomarkers of chronic diseases. Neighbourhood children born normal birth weight (NBW) (>2.5 kg) were enrolled for comparison.

**Results:**

The study included 667 LBW and 87 NBW children. Height-for-age, body mass index for-age (BMIZ), fat-free mass index, and waist circumference of LBW children were lower than those of NBW children. LBW children could jump farther. LBW children who were now overweight had higher leptin, triglyceride and VLDL and lower HDL, compared to NBW children in the same BMIZ category. Currently underweight LBW children had higher adiponectin and lower leptin levels than the reference group. There were no differences between LBW and NBW children in visfatin, fasting glucose and insulin, hemoglobin A1c, triglyceride, low density lipoprotein or C-reactive protein.

**Conclusion:**

At 8–14 years few children were overweight and there were few differences in some risk markers of chronic disease between LBW and NBW children. Overweight, which was associated with some increased risk markers, may increase with age, thus timely counselling and monitoring of these LBW children will be important to mitigate these risks.

## Introduction

Weight at birth is an important predictor of immediate and long-term health (1). The prevalence of low birth weight (LBW) in low- and middle-income countries is more than twice that of high income countries (2). Though repeated rounds of Indian national surveys from 2005 to 06 to 2019-21 have reported a drop in the prevalence of LBW from 22% to 18%, the proportion of children born LBW in India in the past 5 years remains unchanged at 18% (3, 4). LBW not only increases the risk of fetal and neonatal mortality and morbidity but also is associated with impaired physical and cognitive development, thus adding to the burden of the nation's public health (2).

LBW has been associated with the risk of obesity, insulin resistance and cardiometabolic diseases in later life (5). An explanation for this increased risk of non-communicable diseases (NCDs) is the capacity-load model whereby children born LBW start their life with low metabolic capacity, which, starting in childhood, gets challenged by the increasing exposure to metabolic load, as evidenced by overweight (6). This is relevant to a country like India, with a high prevalence of LBW infants, coupled with a nutritional and epidemiological transition, resulting in a surge of diabetes and other NCDs (7, 8). Indians are at an increased risk of NCDs at lower body mass index (BMI) than other ethnic groups (9, 10). It is important to understand the mechanisms involved in this risk.

White adipose tissues produce the hormones leptin, visfatin and adiponectin, collectively called adipokines (11). While leptin and visfatin exert pro-inflammatory properties, adiponectin is anti-inflammatory (12). An increase in leptin and visfatin which reflect body fat mass, and a concomitant decrease in adiponectin level may contribute to the pathogenesis of obesity-linked metabolic and cardiovascular complications (11, 13–16). Plasma adiponectin levels are negatively associated with the accumulation of body fat, especially, visceral fat (13). Low adiponectin is linked to metabolic syndrome and recognized as a standalone risk factor for type 2 diabetes in later life (13, 17).

This study was designed to determine if Indian school-aged children born term LBW differ from those born normal birth weight with respect to their body composition, levels of adipokines, and other biochemical parameters and whether differences depended on children's later anthropometry.

## Materials and methods

The term-born LBW children in the study, were participants of the Delhi Infant Vitamin D Supplementation Study (DIVIDS) cohort (18, 19) set up in 2007. Of the 2079 LBW children in the original DIVIDS cohort (DIVIDS-1), 912 were followed up in DIVIDS-2 (2012-14) (19). This study (referred to as DIVIDS-3), conducted from November 2019 to December 2022, at the Institute of Home Economics (IHE), New Delhi, is a cross-sectional follow-up of DIVIDS children now aged 8–14 years (18, 19) (Supplementary Figure S1).

### Study participants

Full term LBW children belonging to the second follow up study (DIVIDS-2) (19) were traced and contacted using the telephone numbers and addresses documented earlier. They were invited to join the DIVIDS-3 study. For comparison, children born normal birth weight (NBW) (>2.5 to <4 kg) at term (>37 weeks gestation) from the same neighbourhoods and comparable age range (8–14 years) were contacted for enrolment. If a parent of a NBW child showed willingness to participate, the child's inclusion in the study was determined by checking the hospital discharge document having the date of birth, birth weight, and gestational age at birth. Children whose birth weight could not be documented were not recruited.

### Study procedures

Parents and children were invited to IHE for assessments after an overnight fast by the child. Consent from the parent/guardian and assent from the child was obtained. A baseline saliva sample was collected for a deuterium dilution test and a deuterated water dose was given (see below). Details pertaining to sociodemographics and household assets were obtained using a questionnaire and anthropometric measures were taken. A medical doctor conducted a medical check-up and measured blood pressure using a digital OMRON machine (Model No. JPN1).

### Anthropometry

Anthropometric measures, including, height, weight, circumferences of waist, hip and mid-upper arm, and skinfold thickness at triceps and subscapular were taken using standard equipment, following standard operating procedures (20). Standing height (cm) was measured with a stadiometer (model no. SECA 222) to the nearest 0.1 cm. A digital Omron weighing scale (model no. HN-286) accurate to 0.1 kg was used to measure body weight. Circumferences of waist, hip and mid-upper-arm were measured to the nearest 0.1 cm using non-stretch tapes (model no. SECA 201). Triceps and subscapular skinfold thickness were measured by Harpenden skinfold callipers to the nearest 0.2 mm. All measures were taken in triplicate and medians were used in analysis. Technical error of measurement (TEM) was calculated periodically through the period of data collection and was within acceptable limits for all anthropometric measurements (21).

### Muscle strength

The muscle strength of children was measured by hand grip strength for upper limbs and long jump for lower limbs. Hand grip strength was measured with a hand-held dynamometer (TKK 5401, Takei Scientific Instruments Co. Ltd., Japan). Each child also performed a distance jump from a standing start. The distance from the jump-starting line to the trailing edge of the heel at the point of landing was measured. These measurements were taken in triplicate. The highest value attained by the dominant hand for grip strength and the maximum jump distance covered (expressed in metres) were used for analysis.

### Biochemical assessment

The venous blood of the children was collected by a trained phlebotomist in EDTA-coated tubes to determine glucose (hexokinase method; Beckman Coulter AU-680), insulin (chemiluminescence; Architect I-2000), hemoglobin A1c (HbA1c) (HPLC; Tosoh G-8), plain tubes for lipid profile (spectrophotometry; Beckman Coulter AU-680), adipokines (ELISA; Bio-Rad, model no. PR 4100), C-reactive protein (CRP) (immunoturbidimetry; Beckman Coulter AU-680). For all assays except adipokines, blood samples were sent to Dr Lal Path laboratory (Delhi; NABL accredited) for analysis.

Adipokine blood samples were centrifuged at IHE and the serum stored at −80°C for later batched analysis. Leptin and adiponectin were estimated using ELISA kits from Diagnostic Biochem Canada, while visfatin was assessed using kits from BioVendor, R&D. Estimation of all adipokines was done in duplicate. Internal standards were included in each run and results for these were within acceptable limits. Intra- and inter-assay coefficients of variation for a pooled serum sample of adipokines included in each run were within 5% and 10% respectively.

### Deuterium dilution test

After collection of the baseline saliva sample using a cotton swab, a standard dose of 1.8 g deuterated water, equivalent to 0.06 g/kg body weight for an average 30 kg child, was orally administered to each child. Four hours later (equilibrium period), a post-dose saliva sample was collected for analysis of deuterium enrichment. The saliva samples were stored at −80°C and analysed for deuterium enrichment by isotope ratio mass spectrometry at St John's Medical College, Bengaluru, as described previously (19).

### Sample size

The sample size was based on the number of children who could be recruited: 650 LBW and 87 NBW children. Based on calculations for differences between two means, and including a calculation for groups of different sizes (22), the number of children available was sufficient to detect an effect size of 0.11 in unadjusted analyses using a two-sided test with α = 0.05% and 80% power.

### Ethics

Ethical approval was obtained from Institutional Ethical Committee of IHE, University of Delhi, Sitaram Bhartia Institute of Science and Research, and the London School of Hygiene and Tropical Medicine. Parents provided written or thumbprint (among people unable to write) consent for their child's participation and a written assent was obtained from the children themselves. Details pertaining to the participants were kept confidential. Whenever necessary, treatment or referrals were made by the study medical doctor.

### Statistical analysis

Data was double-entered in EpiInfo 7.0.2, cleaned and exported to STATA 16.0 for further analysis. Principal Component Analysis (PCA) was used to compute socio-economic status (SES) using asset ownership data; the first component of the PCA was divided into terciles. Standardised age- and sex specific z-scores for height (HAZ) and BMI (BMIZ) were calculated using Stata zanthro commands and the WHO growth references. Fat mass index (FMI) and fat-free mass index (FFMI) were calculated by dividing fat mass and fat-free mass by the square of height (m), respectively. Chi square test for proportions was used to assess the differences in the sociodemographics of the two groups of children. Analyses for body composition and anthropometric variables were stratified by sex. Biochemical parameters differed little by sex so were analysed with both sexes together and sex as a covariable. Multivariate linear regression determined differences in the continuous outcomes, adjusted for age and sex for biochemical outcomes. Regression analyses were further adjusted for sociodemographic variables that differed between LBW and NBW children. We did not control for the original treatment group in the DIVIDS-1 vitamin D intervention trial from birth to 6 months since we detected no remaining group differences in the children when previously followed up aged about 5 years (19). In order to investigate the capacity-load model, we assessed the interaction between birth weight group and current BMIZ category for their associations with adipokines. Forest plots were generated to illustrate these results.

To account for potential errors in weight measurements in routine healthcare services, we did a sensitivity analysis which included only those NBW children with documented birthweight >2.7 kgs.

## Results

### Sociodemographic characteristics

There were 87 NBW and 667 LBW children. The low enrolment of NBW children was attributable to our inclusion criterion of having a documented birthweight record of the child. Half of the children in each group were girls. The mean birth weight for LBW children was 2.2 (SD 0.1) kg and 2.9 (SD 0.3) kg for NBW children (Table 1). Two-third of fathers and over half of mothers had at least secondary education. Most mothers were housewives and most fathers were daily wagers for both groups of children. More NBW participants were from the medium SES tercile with fewer in the high SES tercile. Mother's education and SES tercile were the only differences between LBW and NBW children and these variables were included in adjusted analyses.

**Table 1:** Socio-demographics of LBW and NBW children.

Parameters		LBW (*n* = 667)	NBW (*n* = 87)	*p*-value*
Birthweight (kg), Mean (SD)		2.2 (0.1)	2.9 (0.3)	<0.001
Females		349 (52.3%)	43 (49.4%)	0.77
Mother's education^a^	None	107 (16%)	5 (5%)	0.01
	Primary	91 (14%)	7 (8%)	
	Middle	166 (25%)	31 (36%)	
	Secondary	232 (35%)	32 (36%)	
	College/technical/university	68 (10%)	12 (14%)	
Mother's occupation^a^	Housewife	541 (81%)	72 (83%)	0.70
	Employed	119 (18%)	15 (17%)	
	Student/other	5 (1%)	0 (0%)	
Father's education	None	42 (6%)	1 (1%)	0.17
	Primary	53 (8%)	5 (6%)	
	Middle	136 (20%)	23 (26%)	
	Secondary	323 (49%)	42 (48%)	
	College/technical/university	100 (15%)	16 (18%)	
	Other/NA	13 (2%)	0 (0%)	
Father's occupation	Self employed	250 (37%)	33 (38%)	0.96
	Daily wager	382 (57%)	50 (57%)	
	Student/other/NA	35 (5%)	4 (4%)	
School type	Government	327 (49%)	41 (47%)	0.70
	Private	331 (50%)	46 (53%)	
	Charitable	6 (1%)	–	
SES tercile	Low	226 (34%)	26 (30%)	0.04
	Medium	212 (32%)	39 (45%)	
	High	229 (34%)	22 (25%)	

LBW, low birth weight; NBW, normal birth weight; NA, not applicable; SES, socio-economic status.

^a^ 3 missing mother's education (LBW); 2 missing mother's occupation (LBW).

* *p*-value was calculated using chi-square for categorical variables, *t*-test for continuous variables and significant at <0.05.

### Anthropometry and body composition

The LBW children were significantly older than the NBW children (Table 2) which likely contributed to the higher values of some anthropometric measures, such as LBW males' height in cm; however, HAZ was lower in these LBW males compared to NBW males. For both sexes, waist circumference was lower in the LBW children and hip circumference was also lower for LBW males than for their NBW counterparts. NBW children had higher FFMI than LBW children. Grip strength did not differ between LBW and NBW children but, even after adjusting for age and height, LBW children could jump further than NBW children. Systolic blood pressure was higher in LBW compared with NBW males in age-adjusted analyses but not in analyses further adjusted for maternal education and SES.

**Table 2:** Anthropometry, body composition, muscle strength and clinical data of LBW and NBW children by group and sex^a^^,^^b^.

	MALE (*n* = 361)					
	LBW (*n* = 318)	NBW (*n* = 43)	Mean difference (95% CI)	*p*-value (adjusted for age)	Mean difference (95% CI)	*p*-value (fully adjusted)^c^
Age (years)	11.2 (0.8)	10.4 (1.5)	**–**	**–**	−0.85 (−1.15, −0.54)	**<0**.**001**
Height (cm)	138.0 (8.5)	136.6 (11)	3.36 (1.10, 5.62)	**0**.**004**	3.79 (1.63, 5.95)	**0**.**001**
HAZ score^d^	−0.9 (0.9)	−0.5 (1.1)	0.43 (0.09, 0.76)	**0**.**01**	0.44 (0.13- 0.75)	**0**.**005**
Stunted (*n*, %)^d^	49 (15.4)	5 (11.4)		0.17		0.47
BMIZ score^d^	−1.07 (1.5)	−0.7 (1.8)	0.40 (−0.11, 0.91)	0.12	0.42 (−0.06, 0.90)	0.089
Grade 3 & 2 thinness (*n*, %)	54 (16.9)	6 (13.9)	**–**	0.16	**–**	0.10
Grade 1 thinness (*n*, %)	112 (35.2)	15 (34.9)				
Normal weight (*n*, %)	132 (41.5)	14 (32.5)				
Overweight/obesity (*n*, %)	20 (6.2)	8 (18.6)				
Waist circumference (cm)^e^	57.5 (8.2)	58.6 (11.3)	3.03 (0.25 5.81)	**0**.**03**	3.45 (0.72, 6.17)	**0**.**01**
Hip circumference (cm)^e^	67.8 (7.6)	68.8 (10.4)	3.55 (1.09, 6.01)	**0**.**005**	3.87 (1.52, 6.23)	**0**.**001**
MUAC (mm)^e^	19.1 (2.9)	19.3 (3.8)	0.89 (−0.08, 1.88)	0.07	1.03 (0.07, 1.99)	**0**.**03**
Triceps skinfold (mm)	9.5 (5.4)	10.1 (7)	1.01 (−0.85, 2.87)	0.28	1.18 (−0.63, 3.01)	0.20
Subscapular skinfold (mm)^e^	9.2 (6.1)	10.4 (8.8)	1.68 (−0.45, 3.82)	0.12	1.83 (−0.28, 3.96)	0.09
Long jump (m)^d^^,^^e^	1.40 (0.2)	1.24 (0.2)	−0.10 (−0.18, −0.03)	**0**.**003**	−0.11 (−0.18, −0.04)	**0**.**002**
Hand grip strength (kg)^e^	13 (3.1)	12.3 (4)	0.63 (−0.29, 1.57)	0.18	0.73 (−0.20, 1.66)	0.12
	Body composition using deuterium dilution test^e^					
Fat mass index	4.2 (3.4, 5.6)	3.8 (3, 5.4)	0.45 (−1.38, 2.29)	0.62	0.46 (−1.29, 2.23)	0.60
Fat free mass index	10.6 (9.8, 11.5)	10.7 (10.1,12.4)	3.28 (1.64, 4.91)	**<0**.**001**	3.38 (1.76, 5.00)	**<0**.**001**
	Blood pressure					
Systolic BP (mm/Hg)	106 (9)	101 (11)	−3.35 (−6.61, −0.09)	**0**.**04**	−2.38 (−5.63, 0.85)	0.14
Diastolic BP (mm/Hg)	64.6 (6.8)	63.2 (7.7)	−1.50 (−3.85, 0.85)	0.21	−1.25 (−3.65, 1.14)	0.30
Pulse (bpm)	85.8 (13.2)	84.5 (12.0)	−0.27 (−4.69, 4.15)	0.90	0.22 (−4.28, 4.73)	0.92

	FEMALE (*n* = 392)					
	LBW (*n* = 349)	NBW (*n* = 43)	Mean difference (95% CI)	*p*-value (adjusted for age)	Mean difference (95% CI)	*p*-value (fully adjusted)^c^
Age (years)	11.3 (0.9)	10.6 (1.7)	**–**	**–**	−0.59 (−0.92, −0.26)	**<0**.**001**
Height (cm)	139.4 (8.7)	135.2 (12.5)	−0.50 (−2.69, 1.68)	0.65	−0.69 (−2.87, 1.47)	0.53
HAZ score^d^	−1.07 (1.0)	−1.1 (0.9)	−0.03 (−0.35, 0.29)	0.85	−0.07 (−0.39, 0.25)	0.66
Stunted (*n*, %)^d^	67 (19.2)	11 (25.6)		0.43		0.40
BMIZ score^d^	−1.0 (1.3)	−0.7 (1.3)	0.45 (0.02, 0.89)	**0**.**03**	0.26 (−0.16, 0.69)	0.23
Grade 3 & 2 thinness (*n*, %)	77 (22)	9 (20.9)	**–**	0.05	**–**	0.09
Grade 1 thinness (*n*, %)	93 (26.7)	8 (18.6)				
Normal weight (*n*, %)	158 (45.2)	21 (48.9)				
Overweight/obesity (*n*, %)	21 (6.0)	5 (11.6)				
Waist circumference (cm)^e^	57.3 (8.4)	58.1 (9.4)	3.11 (0.64, 5.57)	**0**.**01**	2.82 (0.37, 5.27)	**0**.**02**
Hip circumference (cm)^e^	70.8 (9.1)	69.4 (9.8)	1.79 (−0.65, 4.23)	0.15	1.46 (−0.95, 3.88)	0.23
MUAC (mm)^e^	19.4 (3)	19.2 (3.1)	0.69 (−0.17, 1.55)	0.11	0.53 (−0.32, 1.38)	0.22
Triceps skinfold (mm)	11.1 (5.2)	10.6 (4.8)	0.45 (−1.15, 2.05)	0.58	0.17 (−1.40, 1.76)	0.82
Subscapular skinfold (mm)^e^	11.1 (6.3)	11.2 (7.1)	1.41 (−0.55, 3.39)	0.15	1.18 (−0.77, 3.15)	0.23
Long jump (m)^d^^,^^e^	1.20 (0.2)	1.14 (0.2)	−0.07 (−0.14, −0.007)	**0**.**02**	−0.07 (−0.14, 0.0001)	0.05
Hand grip strength (kg)^e^	12.0 (3)	11.2 (3.8)	0.26 (−0.60, 1.14)	0.54	0.15 (−0.72, 1.03)	0.73
	Body composition using deuterium dilution test^e^					
Fat mass index	4.9 (4, 6.6)	4.3 (3.7, 5.6)	0.32 (−1.41, 2.06)	0.71	0.20 (−1.51, 1.92)	0.81
Fat free mass index	10.2 (9.3,11.1)	10.7 (9.6, 11.8)	1.41 (0.02, 2.81)	**0**.**04**	1.30 (−0.06, 2.68)	**<0**.**001**
	Blood pressure					
Systolic BP (mm/Hg)	104 (10)	100 (12)	−1.69 (−5.26, 1.87)	0.35	−2.27 (−5.85, 1.30)	0.20
Diastolic BP (mm/Hg)	63.4 (7.1)	63.8 (8.8)	1.00 (−1.54, 3.54)	0.44	0.84 (−1.72, 3.41)	0.52
Pulse (bpm)	89.4 (14.3)	84.7 (10.4)	−3.92 (−8.87, 1.02)	0.12	−3.87 (−8.89, 1.13)	0.13

HAZ: height for age z-score; BMIZ: body mass index z-score; MUAC: mid-upper-arm circumference; LBW: low birth weight; NBW: normal birth weight.

Bold values are the *p* values that shows significant difference in the variables.

^a^ Mean (SD) or median (25th, 75th percentiles).

^b^ *p* value calculated by linear regression for continuous outcomes and ordinal regression for BMI for age group, chi-square for frequency.

^c^ *p*-value fully adjusted for variables like age, mothers education, socio-economic status.

^d^ *p*-value adjusted for age and height.

^e^ 1 missing waist circumference; 3 missing hip circumferences; 1 missing subscapular skinfold; 6 missing long jump; 1 missing hand grip strength; 31 missing body composition.

### Biochemical tests

LBW children had slightly but significantly higher total cholesterol, very low-density lipoproteins (VLDL) and high-density lipoprotein (HDL) than NBW children (Table 3). In LBW children, serum adiponectin levels were higher while leptin levels were lower than in NBW children, in both unadjusted and adjusted analyses. There were no differences in glucose homeostasis variables (HbA1c, fasting glucose and insulin) or CRP levels among the two groups of children.

**Table 3:** Biochemical data of LBW and NBW children by group^a^.

Parameters	LBW (*n* = 659)	NBW (*n* = 82)	Mean difference (95% CI)	*p*-value^b^	Mean difference (95% CI)	*p*-value^c^
Cholesterol total (mmol/L)	8.4 (1.5)	8.2 (1.6)	−0.39 (−0.73, −0.04)	**0**.**02**	−0.64 (−1.11, −0.16)	**0**.**008**
Triglyceride (mmol/L)^d^	3.9 (3.1, 5.3)	3.8 (3.2, 5.1)	0.21 (−0.26, 0.68)	0.37	0.24 (−0.41, 0.90)	0.47
HDL cholesterol (mmol/L)	2.7 (0.6)	2.6 (0.7)	−0.17 (−0.30, −0.04)	**0**.**01**	−0.24 (−0.43 −0.06)	**0**.**009**
LDL cholesterol (mmol/L)	4.5 (1.2)	4.8 (1.5)	0.24 (−0.04, 0.53)	0.09	0.009 (−0.39, 0.41)	0.96
VLDL cholesterol (mmol/L)	1.1 (0.4)	1.0 (0.4)	−0.13 (−0.22, −0.04)	**0**.**004**	−0.11 (−0.24, 0.01)	0.07
HbA1c (%)	5.4 (0.4)	5.4 (0.3)	−0.02 (−0.10, 0.05)	0.50	−0.05 (−0.16, 0.05)	0.31
Glucose (fasting) (mmol/L)	4.8 (0.4)	4.7 (0.4)	−0.06 (−0.15, 0.02)	0.13	−0.0003 (−0.11, 0.11)	0.99
CRP^e^ <2 (mg/L) >2 & <5 (mg/L) >5 (mg/L)	578 (87.7%) 49 (7.4%) 32 (4.8%)	75 (90.3%) 3 (3.5%) 5 (6%)	–	0.66	–	0.69
Insulin (fasting) (pmol/L)	49.1 (41.4)	48.9 (35.8)	5.49 (−4.25, 15.2)	0.26	7.06 (−6.48, 20.61)	0.30
Adiponectin (µg/ml)^d^^,^^f^	12.3 (6.7, 19.1)	9.7 (5.3, 15.0)	−0.45 (−0.64, −0.26)	**<0**.**001**	−0.43 (−0.70, −0.16)	**0**.**001**
Leptin (ng/ml)^d^^,^^f^	3.2 (1.3, 7.9)	4.0 (1.6, 11.6)	0.37 (0.10, 0.64)	**0**.**006**	0.62 (0.26, 0.98)	**0**.**001**
Visfatin (ng/ml)^d^^,^^f^	0.74 (0.3, 1.3)	0.76 (0.4, 1.3)	0.11 (−0.15, 0.37)	0.40	0.14 (−0.22, 0.51)	0.44

LBW, low birth weight; NBW, normal birth weight; HDL, high density lipoprotein; LDL, low density lipoprotein; VLDL, very low density lipoprotein; CRP, c-reactive protein.

Bold values are the *p* values that shows significant difference in the variables.

^a^ Mean (SD) until specified.

^b^ *p* calculated by linear regression for continuous outcomes and ordinal regression for categorical outcomes, adjusted for age and sex.

^c^ *p*-value calculated by linear regression for continuous outcomes and ordinal regression for categorical outcomes, adjusted for age, sex, mother's education and socioeconomic status tercile.

^d^ Median (25th, 75th percentile).

^e^ *n* (%).

*^f^*p-value calculated using log transformed values.

**Figure 1:**
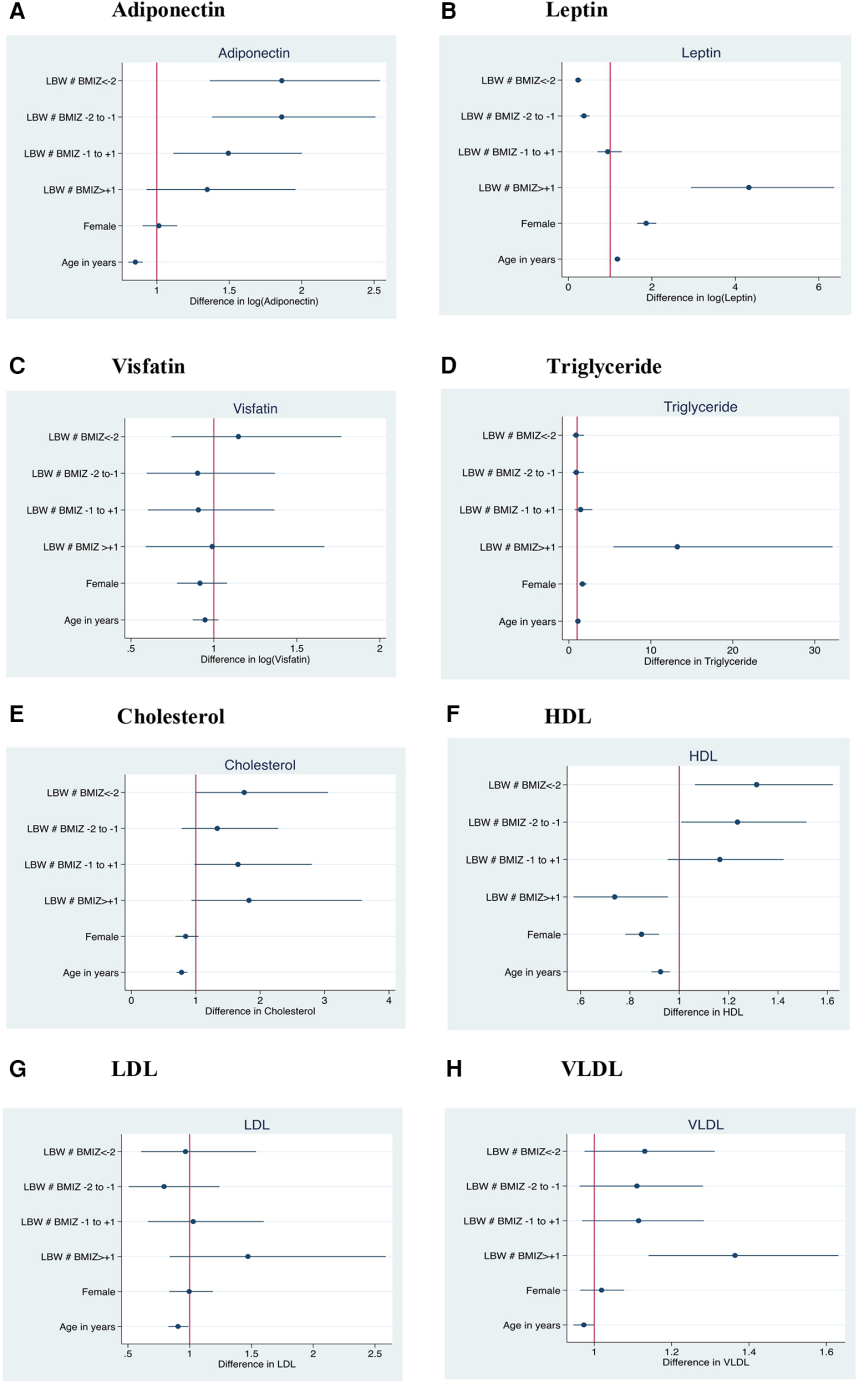
Association of current body mass index with adiponectin, leptin, visfatin, cholesterol, HDL Triglyceride, LDL and VLDL levels in low birth weight compared with normal birth weight children. The reference group was NBW children of currently normal BMIZ; Females are compared to males as the reference; Y axis in the forest plot indicate LBW children within different categories of current BMI; BMIZ < −2: Grade 2 & 3 thinness (*n* = 146); BMIZ −2 to −1: Grade 1 thinness (*n* = 228); BMIZ −1 to +1: Normal (*n* = 325); BMIZ > +1: Overweight and obese (*n* = 54); LBW, low birth weight; BMIZ, body mass index Z score; HDL, high density lipoprotein.

We further investigated whether the associations were modified by current BMI, expressed as categories based on BMIZ scores (Figure 1). Adiponectin was high in all BMI categories of LBW children compared with the reference group of NBW children in the same BMI category. Younger children had lower adiponectin than older children but there was no association with sex. Compared with the reference group, underweight children born LBW had lower leptin levels and overweight children had higher levels; girls had higher leptin levels, independent of BMIZ. Unlike leptin, visfatin levels were higher in undernourished children with BMIZ < −2 and lower in overweight/obese LBW children when compared to their normal counterparts. Total cholesterol was higher among all BMI categories of LBW children. HDL was higher in underweight LBW children and there were trends for higher triglyceride, LDL and VLDL among overweight/obese LBW children when compared to NBW children of similar BMI. HDL levels were lower in girls, while cholesterol, HDL, LDL and VLDL were lower in younger children.

Results of sensitivity analysis, involving NBW children weighing >2.7 kgs at birth (total *N* = 55; Male = 31; Female = 24) were similar to those based on primary analysis including 87 NBW children with >2.5 kg birth weight (Supplementary Tables S1 and S2).

## Discussion

The aims of our study were to assess the impact of early life under-nutrition on health risks in Indian children aged 8–14 years born low birth weight at term and to determine whether any differences were influenced by current anthropometry. Our results indicate that HAZ and BMIZ scores of LBW children, born at term, were lower than those of NBW children while the waist circumference of all LBW children and hip circumference of only LBW male children was lower than the NBW counterparts. Blood biochemistry for assessing markers of chronic NCD risk indicated modest differences: Children born LBW had marginally higher total cholesterol and HDL, compared to NBW children whereas fasting glucose and insulin, HbA1c, triglyceride, LDL and CRP did not differ between the two groups of children. The LBW children had higher plasma adiponectin and lower plasma leptin than the NBW children but there was no difference in visfatin. Birth weight group interacted with current BMI category for associations with biochemical results; specifically, currently overweight LBW children had higher leptin and VLDL but lower HDL than overweight NBW children and underweight LBW children had higher adiponectin than underweight NBW children. Overall, the results suggest metabolic differences between LBW and NBW children; LBW children gain excess weight.

LBW children are more likely to be stunted than NBW (23, 24), which may further reflect in adulthood (25). Reduced adult height has been correlated in some studies with impaired glucose tolerance test (26, 27). Thus, the reduced HAZ in these LBW children could become a risk factor for later life type 2 diabetes. Moreover, muscle being a crucial site for insulin-stimulated glucose absorption, the lower FFMI in the LBW children may increase their future risk of insulin resistance (10). In addition, increased consumption of an energy-dense diet, primarily from fat, in these LBW children (27) can predispose them to obesity later in life which could then help to explain why adults with LBW have higher percentages of body fat (28). The low anthropometric variables seen in most DIVIDS LBW children may be because of their ongoing adverse socioeconomic conditions (29, 30). Although some studies of long-term outcomes associated with LBW have not mentioned gestational age (31, 32), Yajnik et al. (33) showed that the risk of NCDs in later life could prevail, irrespective of the gestational age of the LBW child.

Persistently high serum leptin levels as seen in obesity, may lead to the development of leptin resistance (11, 34). In our study, only those LBW children with higher current BMIZ score, although still low with respect to the international standards, showed higher leptin levels, perhaps because of increased adiposity. Obese individuals often have not only higher leptin levels but also higher visfatin and lower adiponectin levels relative to lean persons (35–37). In a case-control study from South America, full term LBW children (*n* = 765; 6–11 years old) with high abdominal circumference, insulin resistance, and systolic blood pressure had lower circulating adiponectin concentration (38). Additionally, European female adolescents born LBW (*n* = 429) had high fasting serum leptin levels (39). The BMI of the study population in the above-mentioned studies showed no correlation with circulating levels of adipokines. The difference from these studies and ours may be that in these studies participants were in more socioeconomically advantaged environments which permitted increases in total and visceral fat.

While the LBW children had higher serum cholesterol overall as well as higher leptin, VLDL and triglyceride compared to NBW children of similar BMI, and exhibited lower FFMI, changes in markers of risk of chronic disease in our cohort were fairly modest, possibly because these children are still young; changes in these risk markers are usually manifested in adulthood. From clinical and public health perspectives, stakeholders should be aware of a greater possibility of developing dyslipidemia and associated cardiometabolic risk factors like higher blood pressure in term LBW adolescents, especially if they gain excess weight.

The strength of this study is the anthropometric measurements, body composition, and biochemical profile of a large number of school-going Indian children born LBW weight for whom we have previous data. A limitation is that we were only able to enrol a limited number of normal birth weight children (*n* = 87) with birth weight >2.5 kg.

## Conclusion

This study adds evidence to the growing literature on anthropometric and blood profiles of LBW term Indian children during childhood. Anthropometric or biochemical risk factors might not be evident in childhood but may surface in later adult life (28). The findings of higher leptin and triglycerides among LBW with high BMIZ score compared to NBW children of similar BMI suggests a potential future problem which requires intervention during childhood when risks are potentially modifiable. In the backdrop of rapidly escalating NCD burden, there is an urgent need to formulate appropriate guidelines for routine screening for cardiometabolic risk and life-style counselling in adolescents. Future research should focus on identifying robust predictors of developing cardiometabolic risk in adolescence, including from early life antecedents.

## Data Availability

The datasets presented in this article are not readily available due to Government of India restrictions. Requests to access the datasets should be directed to the Principal Investigator (PI), who could apply to the Health Ministry's Screening Committee (HMSC) for requisite permissions for sharing the data.

## References

[1] Alemayehu GM Chernet AG Dumga KT. Determinants of child size at birth and associated maternal factor in gurage zone. J Reprod Infertil. (2020) 21(2):138–45. 32500017 PMC7253936

[2] United Nations Children’s Fund (UNICEF), World Health Organisation (WHO). UNICEFWHO Low birthweight estimates: Levels and trends 2000–2015 (2019). Available online at: https://www.unicef.org/media/96976/file/UNICEF-WHO-Low-Birthweight-estimates-20002015.pdf (accessed June 26, 2023).

[3] International Institute for Population Sciences (IIPS) and MoHFW. National Family Health Survey (NFHS-4), 2015-16: India (2017). Available online at: http://rchiips.org/nfhs/nfhs4Reports/India.pdf (accessed June 21, 2023).

[4] International Institute for Population Sciences (IIPS) and ICF. National Family Health Survey (NFHS-5), 2019-21: India (2021). Available online at: https://dhsprogram.com/pubs/pdf/FR375/FR375.pdf (accessed May 1, 2023).

[5] Kelishadi R Haghdoost AA Jamshidi F Aliramezany M Moosazadeh M. Low birthweight or rapid catch-up growth: which is more associated with cardiovascular disease and its risk factors in later life? A systematic review and cryptanalysis. Paediatr Int Child Health. (2015) 35(2):110–23. 10.1179/2046905514Y.0000000136 25034799

[6] Wells JCK. The capacity-load model of non-communicable disease risk: understanding the effects of child malnutrition, ethnicity and the social determinants of health. Eur J Clin Nutr. (2018) 72(5):688–97. 10.1038/s41430-018-0142-x 29748656

[7] Misra A Singhal N Sivakumar B Bhagat N Jaiswal A Khurana L. Nutrition transition in India: secular trends in dietary intake and their relationship to diet-related non-communicable diseases. J Diabetes. (2011) 3(4):278–92. 10.1111/j.1753-0407.2011.00139.x 21649865

[8] Yajnik CS. Obesity epidemic in India: intrauterine origins? Proc Nutr Soc. (2004) 63(3):387–96. 10.1079/pns2004365 15373948

[9] Misra A Chowbey P Makkar BM Vikram NK Wasir JS Chadha D Consensus statement for diagnosis of obesity, abdominal obesity and the metabolic syndrome for Asian Indians and recommendations for physical activity, medical and surgical management. J Assoc Physicians India. (2009) 57:163–70. 19582986

[10] Thomas N Grunnet LG Poulsen P Christopher S Spurgeon R Inbakumari M Born with low birth weight in rural southern India: what are the metabolic consequences 20 years later? Eur J Endocrinol. (2012) 166(4):647–55. 10.1530/EJE-11-0870 22250073

[11] Li S Li X. Leptin in normal physiology and leptin resistance. Sci Bull. (2016) 61(19):1480–8. 10.1007/s11434-015-0951-4

[12] Zorena K Jachimowicz-Duda O Ślęzak D Robakowska M Mrugacz M. Adipokines and obesity. Potential link to metabolic disorders and chronic complications. Int J Mol Sci. (2020) 21(10):3750. 10.3390/ijms21103570 32443588 PMC7278967

[13] Ohashi K Shibata R Murohara T Ouchi N. Role of anti-inflammatory adipokines in obesityrelated diseases. Trends Endocrinol Metab. (2014) 25(7):348–55. 10.1016/j.tem.2014.03.009 24746980

[14] Challa AS Evagelidou EN Cholevas VI Klortsis DN Giapros VI Drougia AA Growth factors and adipocytokines in prepubertal children born small for gestational age. Diabetes Care. (2009) 32(4):714–9. 10.2337/dc08-1570 19131467 PMC2660477

[15] Dakroub A Nthasser SA Younis N Bhagani H Al-Dhaheri Y Pintus G Visfatin: a possible role in cardiovasculo-metabolic disorders. Cells. (2020) 9(11):2444. 10.3390/cells9112444 33182523 PMC7696687

[16] Khan M Joseph F. Adipose tissue and adipokines: the association with and application of adipokines in obesity. Scientifica (Cairo). (2014) 2014:1–7. 10.1155/2014/328592 25309775 PMC4182896

[17] Hong X Zhang X You L Li F Lian H Wang J Association between adiponectin and newly diagnosed type 2 diabetes in population with the clustering of obesity, dyslipidaemia and hypertension: a cross-sectional study. BMJ Open. (2023) 13(2):060377. 10.1136/bmjopen-2021060377 PMC9972409 36828662

[18] Kumar GT Sachdev HS Chellani H Rehman AM Singh V Arora H Effect of weekly vitamin D supplements on mortality, morbidity, and growth of low birthweight term infants in India up to age 6 months: randomised controlled trial. Br Med J. (2011) 342(7810):2975. 10.1136/bmj.d2975 21628364 PMC3104477

[19] Trilok-Kumar G Kaur M Rehman AM Arora H Rajput MM Chugh R Effects of vitamin D supplementation in infancy on growth, bone parameters, body composition and gross motor development at age 3–6 years: follow-up of a randomized controlled trial. Int J Epidemiol. (2015) 44(3):894–905. 10.1093/ije/dyv116 26130740

[20] Gibson RS. Principles of Nutritional Assessment. 2nd ed. USA: Oxford University Press (2005).

[21] Ulijaszek SJ Kerr DA. Anthropometric measurement error and the assessment of nutritional status. Br J Nutr. (1999) 82(3):165–77. 10.1017/s0007114599001348 10655963

[22] Kirkwood B. Essentials of Medical Statistics. Oxford: Blackwell (1988).

[23] Association of Southeast Asian Nations (ASEAN), United Nations Children’s Fund. (UNICEF) and World Health Organisation (WHO). Regional Report on Nutrition Security in ASEAN, Volume 2. Bangkok; UNICEF (2016). Available online at: https://www.asean.org/wpcontent/uploads/2016/03/Regional-Report-on-Nutrition-Security-in-ASEAN-Volume-2.pdf (accessed October 10, 2023).

[24] Christian P Lee SE Donahue Angel M Adair LS Arifeen SE Ashorn P Risk of childhood undernutrition related to small-for-gestational age and preterm birth in low- and middle-income countries. Int J Epidemiol. (2013) 42(5):1340–55. 10.1093/ije/dyt109 23920141 PMC3816349

[25] Victora CG Adair L Fall C Hallal PC Martorell R Richter L Maternal and child undernutrition: consequences for adult health and human capital. Lancet. (2008) 371(9609):340357. 10.1016/S0140-6736(07)61692-4 18206223 PMC2258311

[26] Brown DC Byrne CD Clark PMS Cox BD Day NE Hales CN Height and glucose tolerance in adult subjects. Diabetologia. (1991) 34(7):531–3. 10.1007/BF00403292 1916060

[27] Trilok-Kumar G Malik A Gusain Y Millerot E Pathak R Filteau S. Dietary patterns of Indian school-aged children and associations with markers of chronic disease risk. Food Sci Nutr. (2023) 11:1–10. 10.1002/fsn3.3631 37970425 PMC10630838

[28] Singhal A Wells J Cole T Fewtrell M Lucas A. Programming of lean body mass: a link between birth weight, obesity, and cardiovascular disease? Am J Clin Nutr. (2003) 77(3):726–30. 10.1093/ajcn/77.3.726 12600868

[29] Rahman MS Howlader T Masud MS Rahman ML. Association of low-birth weight with 415 malnutrition in children under five years in Bangladesh: do mother’s education, socio-economic status, and birth interval matter? PLoS One. (2016) 11(6):e0157814. 10.1371/journal.pone.0157814 27355682 PMC4927179

[30] Abbas F Kumar R Mahmood T Somrongthong R. Impact of children born with low birth weight on stunting and wasting in Sindh province of Pakistan: a propensity score matching approach. Sci Rep. (2021) 11(1):19932. 10.1038/s41598-021-98924-7 34620917 PMC8497567

[31] Dos Santos Alves PJ Araujo Júnior E Henriques ACPT Carvalho FHC. Poderia o baixo peso ao nascimento ser associado com risco cardiovascular na adolescência? Rev Bras Ginecol Obstet. (2016) 38(4):189–95. 10.1055/s-0036-1583171 27128340 PMC10309411

[32] Whincup P Cook D Adshead F Taylor S Walker M Papacosta O Childhood size is more strongly related than size at birth to glucose and insulin levels in 10–11-year-old children. Diabetologia. (1997) 40(3):319–26. 10.1007/s001250050681 9084971

[33] Yagnik CS Fall CHD Vaidya U Pandit AN Bavdekar A Bhat DS Fetal growth and glucose and insulin metabolism in four-year-old Indian children. Diabet Med. (1995) 12(4):330–6. 10.1111/j.1464-5491.1995.tb00487.x 7600749

[34] Myers MG Heymsfield SB Haft C Kahn BB Laughlin M Leibel RL Challenges and opportunities of defining clinical leptin resistance. Cell Metab. (2012) 15(2):150–6. 10.1016/j.cmet.2012.01.002 22326217 PMC3281561

[35] Thiruvengadam V Amperayani S Babu RP Uppuluri R. Correlation of childhood obesity and related insulin resistance with leptin and retinol binding protein 4. Indian J Pediatr. (2015) 82(9):799–804. 10.1007/s12098-015-1706-6 25708059

[36] Mastroeni SS Mastroeni MF Gonçalves MC Debortoli G da Silva NN Bernal RT Cardiometabolic risk markers of normal weight and excess body weight in Brazilian adolescents. Appl Physiol Nutr Metab. (2016) 41(6):659–65. 10.1139/apnm-2015-0632 27227571

[37] Winer JC Zern TL Taksali SE Dziura J Cali AMG Wollschlager M Adiponectin in childhood and adolescent obesity and its association with inflammatory markers and components of the metabolic syndrome. J Clin Endocrinol Metab. (2006) 91(11):4415–23. 10.1210/jc.2006-0733 16926246

[38] Strufaldi MWL Puccini RF Silvério OMA Do Pinho Franco MC. Association of adipokines with cardiovascular risk factors in low birth weight children: a case-control study. Eur J Pediatr. (2013) 172(1):71–6. 10.1007/s00431-012-1846-x 23015046

[39] Labayen Goñi I Ruiz JR Huybrechts I Ortega FB Rodríguez G DeHenauw S Sexual dimorphism in the early life programming of serum leptin levels in European adolescents: the HELENA study. J Clin Endocrinol Metab. (2011) 96(8):E1330–4. 10.1210/jc.20111036 21697251

