# Supplementary material for: Markers of chronic disease risk in term low birthweight Indian children aged 8–14 years
Source: Front Pediatr. 2024 Aug 29;12:1339808. doi: 10.3389/fped.2024.1339808 (PMC11390577; doi:10.3389/fped.2024.1339808)
Supplement: Supplementary file 1 [file Datasheet1.pdf]

Supporting Information

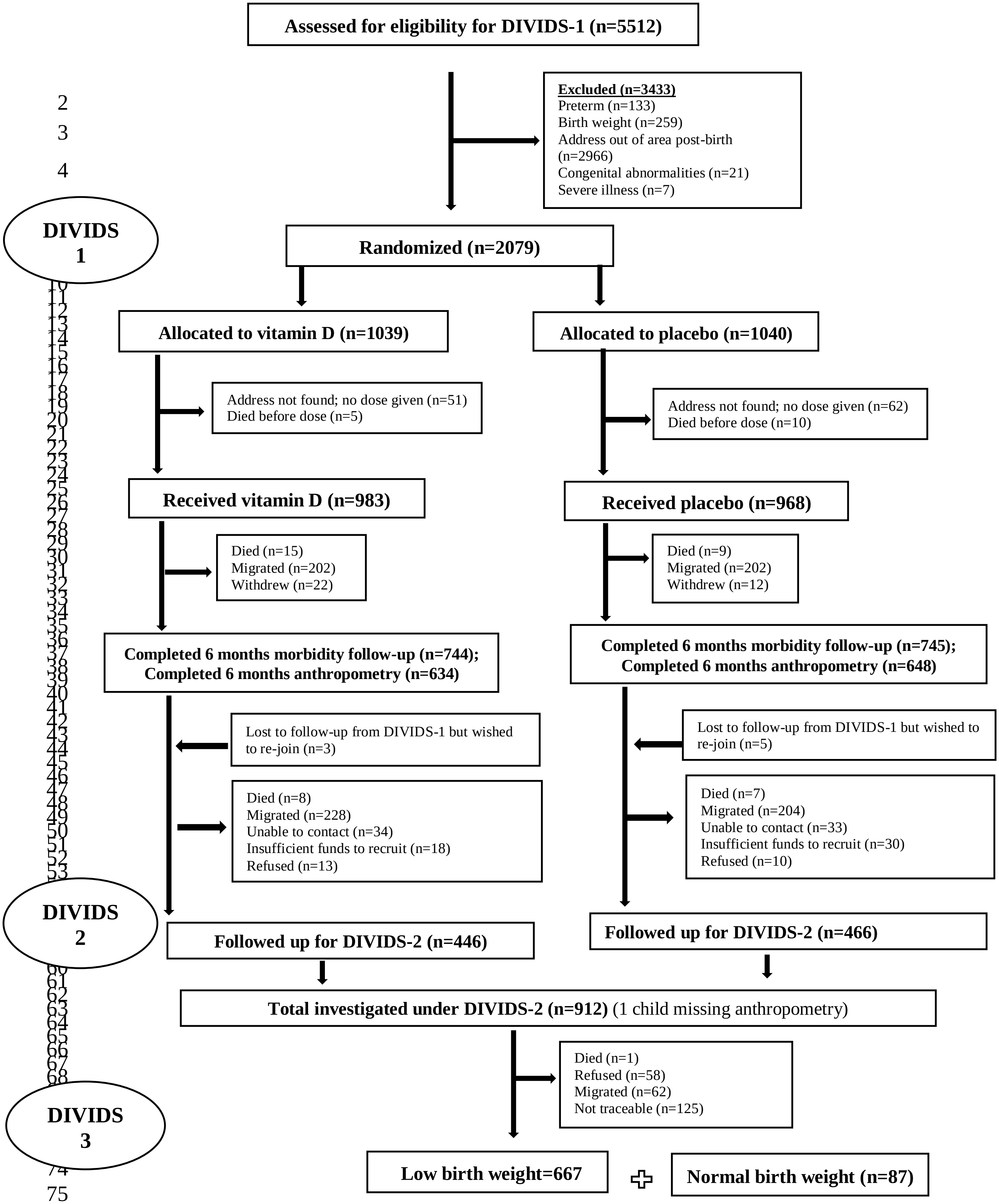

Supplementary Figure 1. Delhi Infant Vitamin D Supplementation (DIVIDS) Study Flowchart

Supplementary Table 1. Anthropometric data of LBW and NBW children by group and sex[a,b]

	MALE (n=349)			FEMALE (n=373)		
	**LBW (n=318)**	**NBW (n=31)**	**p-value (Adjusted for age)**	**LBW (n=349)**	**NBW (n=24)**	**p-value (Adjusted for age)**
Age (years)	11.2 (0.8)	10.5(1.7)	-	11.3 (0.9)	10.9 (1.7)	-
Height (cm)	138.0(8.5)	139.3 (10.6)	**<0.001**	139.4 (8.7)	136.3 (12.8)	0.55
HAZ score[d]	-0.9 (0.9)	-0.27 (1.1)	**<0.001**	-1.07(1.0)	-1.16 (0.8)	0.73
Stunted (n, %)	49 (15.4)	5 (16.3)	0.80	67 (19.2)	5 (20.8)	0.91
BMIZ score	-1.07 (1.5)	-0.43(1.9)	**0.02**	-1.0 (1.3)	-0.57(1.21)	**0.06**
Grade 3 & 2 thinness (n, %)	54 (17)	4 (12.9)	**0.23**	77 (22)	4 (16.6)	**0.24**
Grade 1 thinness (n, %)	112 (35.2)	9 (29)		93 (26.6)	3 (12.5)	
Normal weight (n, %)	132 (41.5)	11 (35.4)		158 (45.2)	15 (62.5)	
Overweight/obesity (n, %)	20 (6.3)	7 (22.7)		21 (6.2)	2 (8.4)	
Waist circumference (cm)[c]	57.5 (8.2)	60.7 (12.3)	**0.004**	57.3 (8.4)	58.6 (8.9)	0.10
Hip circumference (cm)[c]	67.8 (7.6)	71 (11.2)	**<0.001**	70.8 (9.1)	70 (9.8)	0.23
MUAC (mm)	19.1 (2.9)	20 (4.1)	**0.012**	19.4 (3)	19.7 (2.9)	0.18
Triceps skinfold (mm)	9.5 (5.4)	10.8 (7.7)	0.13	11.1 (5.2)	11.2 (5.0)	0.54
Subscapular skinfold (mm)[c]	9.2 (6.1)	11.7(9.9)	**0.02**	11.1 (6.3)	11.2 (6.2)	0.51
Long jump (m) [c,d]	1.40 (0.2)	1.30 (0.2)	0.19	1.20 (0.2)	1.17 (0.2)	0.26
Hand grip strength (kg)[c]	13 (3.1)	13.2 (4)	**0.01**	12.0 (3)	11.8 (3.8)	0.46
Body composition using Deuterium dilution test[c]						
Fat mass index	4.2 (3.4, 5.6)	3.2 (3, 6.4)	0.97	4.9 (4, 6.6)	4.6(4, 6)	0.57
Fat free mass index	10.6 (9.8, 11.5)	11.4 (10.4, 12.7)	**<0.01**	10.2 (9.3,11.1)	10.7 (9.8, 11.8)	**0.01**
Systolic BP (mm/Hg)	105.5 (9.4)	102.4 (11.7)	0.20	103.5 (10.1)	103.9 (10.7)	0.57
Diastolic BP (mm/Hg)	64.6 (6.8)	63.8 (7.8)	0.54	63.4 (7.1)	67.5 (8.7)	**0.007**
Pulse (bpm)	85.8 (13.2)	85.82(12.2)	0.79	89.4 (14.3)	89 (8.7)	0.94

LBW: low birth weight; NBW: normal birth weight; HAZ: height for age z score; BMIZ: body mass index z score; MUAC: mid upper arm circumference; BP: blood pressure; [a]mean (SD) or median (25th, 75th percentiles); [b]p-value calculated by linear regression for continuous outcomes and ordinal regression for BMI for age group, chi square for frequency (%); [c]one missing waist circumference; three missing hip circumferences; one missing subscapular skinfold; six missing long jump; one missing hand grip strength; thirty-one missing body composition; [d]p-value adjusted for age and height

Supplementary Table 2. Biochemical data of LBW and NBW children by group[a]

Parameters	LBW (n=659)	NBW (n=52)	p-value[b]
Cholesterol total (mmol/l)	8.4 (1.5)	8.2 (1.6)	0.24
Triglyceride (mmol/l)[c]	3.9 (3.1, 5.3)	3.8 (3.2, 5.2)	0.32
HDL cholesterol (mmol/l)	2.7 (0.6)	2.6 (0.6)	**0.01**
LDL cholesterol (mmol/l)	4.5 (1.2)	4.8 (1.4)	0.20
VLDL cholesterol (mmol/l)	1.1 (0.4)	1.0 (0.4)	**0.05**
HbA1c (%)	5.4 (0.4)	5.4 (0.2)	0.89
Glucose (fasting) (mmol/l)	4.8 (0.4)	4.7 (0.4)	0.17
CRP<2 (mg/l)[d]	578 (88%)	46 (87%)	0.77
CRP >2 &<5 (mg/l)[d]	49 (7%)	3 (6%)	
CRP>5 (mg/l)[d]	32 (5%)	4 (7%)	
Insulin (fasting) (pmol/l)	49.1 (41.4)	52.44 (41.5)	0.19
Adiponectin (µg/ml)[c,e]	12.3 (6.7, 19.1)	10.1 (6.3, 14.6)	**0.001**
Leptin (ng/ml)[c,e]	3.2 (1.3, 7.9)	4.6 (1.7, 14.2)	**0.002**
Visfatin (ng/ml)[c,e]	0.74 (0.3, 1.3)	0.8 (0.5, 1.4)	0.16

LBW: low birth weight; NBW: normal birth weight; CRP: c-reactive protein; HDL: high density lipoprotein; LDL: low density lipoprotein; VLDL: very low density lipoprotein;[a]mean (SD) until specified; [b]p-value calculated by linear regression for continuous outcomes and ordinal regression for categorical outcomes, adjusted for age and sex; [c]median (25th, 75th percentile); [d]n (%); [e]p-value calculated using log transformed values
